# Acquired *EML4-ALK* fusion and *BRAF* V600G dual bypass activation following osimertinib resistance in lung adenocarcinoma and successful treatment with lorlatinib: a case report

**DOI:** 10.3389/fonc.2026.1919298

**Published:** 2026-08-12

**Authors:** Rui Zhang, Jing Li

**Affiliations:** Department of Radiation Oncology, The Fourth Hospital of Hebei Medical University, Shijiazhuang, Hebei, China

**Keywords:** BRAF V600G, EML4-ALK fusion, lorlatinib, non-small cell lung cancer, osimertinib resistance

## Abstract

**Background:**

While osimertinib is the standard of care for *EGFR*-mutated advanced non-small cell lung cancer (NSCLC), acquired resistance is inevitable. Resistance mechanisms involving the simultaneous activation of dual bypass pathways, coupled with the complete loss of the primary *EGFR* mutation, are exceptionally rare and pose significant therapeutic challenges.

**Case presentation:**

We report a clinically instructive case of a 64-year-old female with stage IV lung adenocarcinoma initially harboring an *EGFR* exon 19 deletion. She received uninterrupted first-line osimertinib for approximately five years, with good tolerability and no dose reduction or treatment interruption. During follow-up, a small asymptomatic central nervous system (CNS) oligoprogressive lesion was initially managed with continued osimertinib and close brain magnetic resonance imaging (MRI) surveillance because extracranial disease remained controlled. CyberKnife stereotactic radiosurgery (SRS) was subsequently performed after lesion enlargement, followed by systemic thoracic progression. A repeat biopsy, analyzed via immunohistochemistry and comprehensive next-generation sequencing, revealed a molecularly distinct clonal shift characterized by the simultaneous emergence of an *EML4-ALK* fusion and a *BRAF* V600G mutation, accompanied by the complete clearance of the original *EGFR* mutation. Confronted with this unique clonal evolution, the patient was transitioned to monotherapy with the third-generation ALK tyrosine kinase inhibitor (TKI) lorlatinib. This targeted intervention was associated with rapid regression of the thoracic lesions, while a reduction in the previously irradiated intracranial lesion was also observed.

**Conclusion:**

To our knowledge, this is the first documented case of concurrent acquisition of an *EML4-ALK* fusion and a *BRAF V600G* mutation after five years of first-line osimertinib therapy, in which the *ALK* fusion was prioritized as the predominant actionable driver. This case highlights the importance of longitudinal re-biopsy and comprehensive molecular profiling to distinguish conventional EGFR-TKI resistance from molecularly distinct clonal replacement or oncogenic driver switching after prolonged targeted therapy.

## 1 Introduction

Lung cancer is the leading cause of cancer-related mortality worldwide, with non-small cell lung cancer (NSCLC) accounting for approximately 85% of all cases (1). Among these, mutations in the epidermal growth factor receptor (*EGFR*) gene represent the most common oncogenic drivers, particularly in lung adenocarcinoma. As a third-generation EGFR tyrosine kinase inhibitor (EGFR-TKI), osimertinib has become the standard first-line treatment for *EGFR*-mutated advanced NSCLC, owing to its robust efficacy and high central nervous system (CNS) penetration (2). Nevertheless, despite durable initial clinical benefits, the invariable emergence of acquired resistance poses a substantial hurdle to prolonged patient survival. The mechanisms driving resistance to osimertinib are highly complex and heterogeneous, including on-target secondary mutations (e.g., *EGFR* C797S), activation of bypass signaling pathways (e.g., *MET* or *HER2* amplification), and histologic transformation (3–5). Intriguingly, anaplastic lymphoma kinase (*ALK*) fusions—canonical oncogenic drivers traditionally considered mutually exclusive with *EGFR* alterations—have been sporadically identified as a rare bypass resistance mechanism following EGFR-TKI therapy (6–8). Such events are frequently under-recognized in routine clinical practice, and the underlying biology of *ALK* rearrangements driving osimertinib resistance remains poorly elucidated. This complexity is further compounded by atypical alterations such as the *BRAF* V600G mutation, whose specific role and prognostic value in NSCLC resistance remain undefined. Ultimately, the emergence of these rare and concurrent genetic alterations significantly complicates subsequent therapeutic sequencing.

Herein, we describe the case of a patient with advanced lung adenocarcinoma, initially positive for an *EGFR* exon 19 deletion, a *TP53* mutation, and *EGFR* amplification, who developed acquired resistance to first-line osimertinib after five years of treatment. Subsequent molecular profiling via re-biopsy uncovered a profound genetic shift characterized by the emergence of an *EML4-ALK* fusion and a *BRAF* V600G co-mutation, alongside the complete loss of the original *EGFR* exon 19 deletion clone. The patient subsequently showed rapid regression of thoracic lesions after transition to the third-generation ALK-TKI lorlatinib, while reduction of the previously irradiated intracranial lesion was also observed. To the best of our knowledge, this is the first reported instance of an *EGFR*-mutant NSCLC undergoing an *ALK* rearrangement coupled with a *BRAF* V600G mutation as an osimertinib resistance mechanism. This unique case not only unveils an exceptionally rare pattern of clonal evolution in the context of EGFR-TKI resistance but also underscores the necessity of longitudinal, comprehensive genomic profiling at progression. Such dynamic assessments are essential for uncovering novel resistance mechanisms and guiding precise subsequent therapeutic interventions.

## 2 Case presentation

In January 2021, a 64-year-old female never-smoker presented with a progressively worsening non-productive cough, accompanied by chest tightness and dyspnea. Chest computed tomography (CT) scan revealed a massive right-sided pleural effusion and atelectasis of the right middle and lower lobes. Following therapeutic thoracentesis at a local hospital, approximately 4,000 mL of hemorrhagic pleural fluid was drained. Post-drainage CT scan showed a mass in the right hilum and lower lobe, multiple small right pleural nodules, and enlarged, partially confluent lymph nodes in the mediastinum and right hilum indicative of metastases. Additionally, hyperdense lesions suggestive of bone metastasis were observed in the lower thoracic and lumbar vertebrae. Thoracoscopy revealed multiple yellow-white nodules on the parietal pleura, predominantly distributed on the posterolateral aspect. While brain magnetic resonance imaging (MRI) showed no signs of metastasis, a whole-body bone scan confirmed multiple vertebral metastases. Pathological examination of the pleural biopsy confirmed poorly differentiated lung adenocarcinoma. Based on these findings, the patient was diagnosed with stage IV right lung adenocarcinoma. Tissue-based genomic profiling identified an *EGFR* exon 19 deletion (p.Glu746_Ala750del; variant allele frequency [VAF]: 57.5%), a *TP53* mutation, and *EGFR* amplification. Notably, no *ALK* fusion or *BRAF* mutation was detected by the baseline next-generation sequencing (NGS) panel. The patient was promptly initiated on targeted therapy with oral osimertinib (80 mg once daily), achieving partial response (PR) within one month, per RECIST criteria. Thereafter, osimertinib was continued without dose reduction or treatment interruption, and the treatment was well tolerated. Radiological follow-up evaluations performed at intervals of approximately 3–6 months confirmed durable disease control.

**Figure 1:**
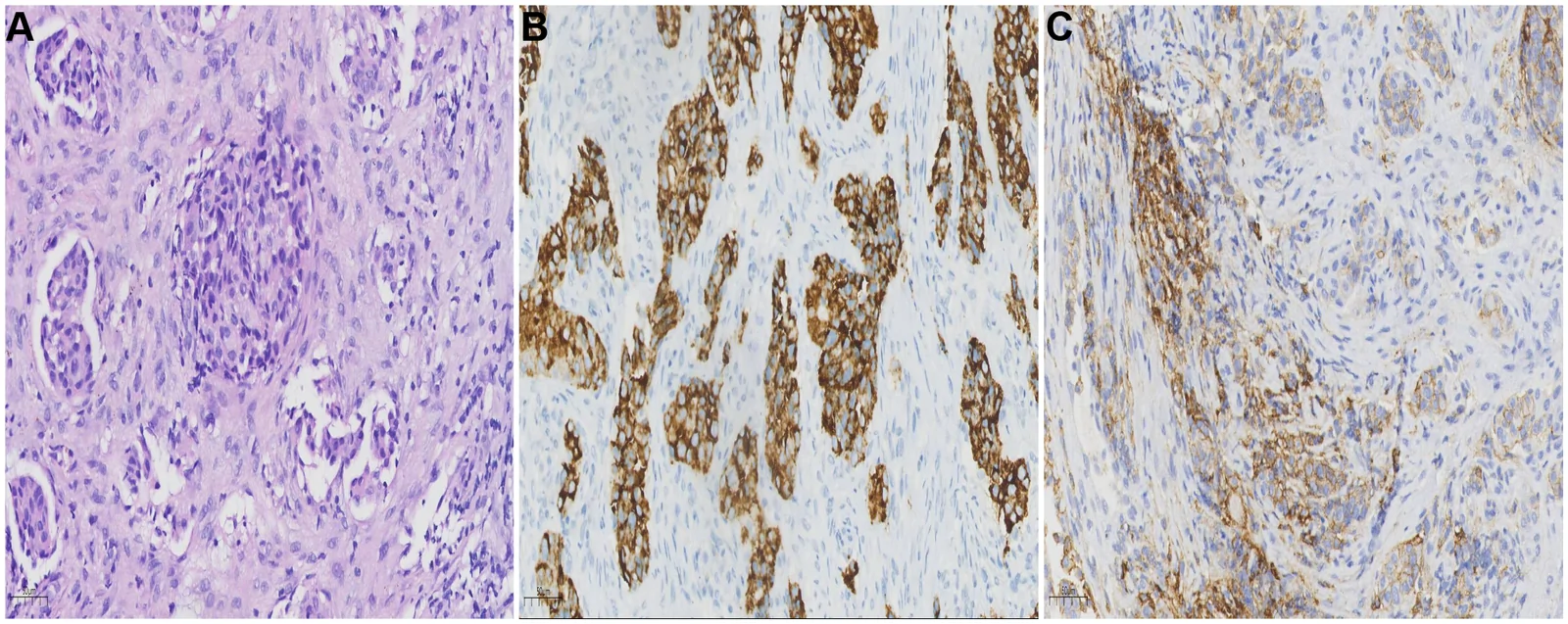
Histopathological and immunohistochemical findings from the repeat biopsy at acquired resistance (March 2026). **(A)** Hematoxylin and eosin (H&E) staining revealing persistent poorly differentiated lung adenocarcinoma, without evidence of histologic transformation. **(B)** ALK immunohistochemistry (IHC) using the VENTANA D5F3 assay showing strong and diffuse granular cytoplasmic staining, validating the acquired *ALK* rearrangement at the protein level. **(C)** PD-L1 IHC using the DAKO 22C3 pharmDx assay. The image exhibits distinct partial or complete membranous staining in viable tumor cells, corresponding to a tumor proportion score (TPS) of 40%. Scale bars = 50 μm.

**Figure 2:**
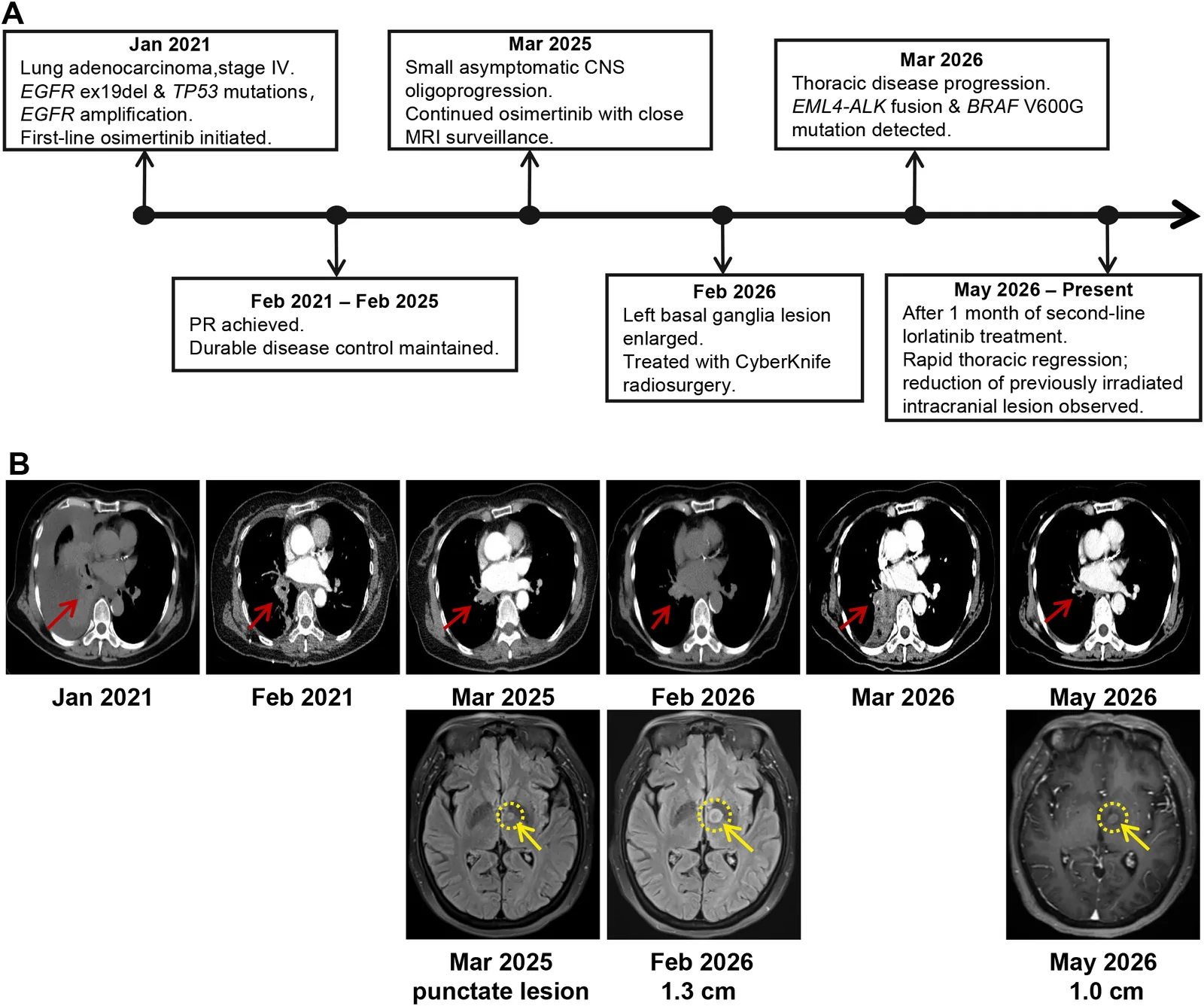
Clinical trajectory and radiological evaluations. **(A)** Timeline of the diagnostic and therapeutic process for the patient. **(B)** Representative serial chest computed tomography (CT) and brain magnetic resonance imaging (MRI) scans, illustrating dynamic changes in thoracic and intracranial lesions during sequential treatment with osimertinib and lorlatinib. Serial chest CT and brain MRI images were selected from the closest available comparable anatomical levels and from available sequences that most clearly demonstrated the target lesions. Minor differences in image appearance reflect inter-institutional acquisition variability. Red arrows indicate thoracic target lesions, whereas yellow arrows and dashed circles indicate the left basal ganglia lesion. Maximal lesion diameters are annotated where reliably measurable.

In March 2025, a follow-up brain MRI revealed a newly developed, asymptomatic enhancing lesion in the left basal ganglia, suggestive of a brain metastasis. At that time, the patient remained neurologically asymptomatic, and extracranial disease was still well controlled on osimertinib. After multidisciplinary discussion and shared decision-making, and in consideration of the patient’s strong preference, continued osimertinib (80 mg once daily) with close brain MRI surveillance was selected because the lesion was small, asymptomatic, and consistent with limited CNS oligoprogression. However, a subsequent contrast-enhanced brain MRI in February 2026 demonstrated enlargement of the left basal ganglia lesion to approximately 1.3 cm in diameter. Consequently, the patient underwent CyberKnife stereotactic radiosurgery (SRS) for the brain metastasis in February 2026 (total dose: 35 Gy in 5 fractions), while continuing osimertinib concurrently. One month later, although a follow-up brain MRI showed stable intracranial disease, a chest CT revealed enlargement of the right hilar and lower lobe masses. Bronchoscopy demonstrated luminal narrowing and mucosal irregularity in the right middle and lower lobes, and a subsequent biopsy pathologically confirmed disease progression of lung adenocarcinoma. Immunohistochemistry revealed a PD-L1 (DAKO 22C3) tumor proportion score (TPS) of 40%, strong positivity for EGFR (90%) and TROP2 (90%), and positive ALK (VENTANA D5F3) expression (**Figure 1**). Repeat targeted NGS of the tumor tissue identified a novel *EML4-ALK* (E14:A20) rearrangement (variant allele frequency [VAF]: 31.96%) and a *BRAF* exon 15 p.V600G missense mutation (c.1799T>G, p.Val600Gly; VAF: 13.57%). Notably, the original *EGFR* exon 19 deletion was completely undetectable. Based on these findings, the patient was transitioned to the third-generation ALK-TKI lorlatinib (100 mg once daily) in April 2026. One month after initiating lorlatinib, follow-up imaging demonstrated significant regression of the pulmonary lesions and mediastinal lymph nodes. A reduction in the previously irradiated intracranial lesion was also observed (**Figure 2**).

## 3 Discussion

With advancements in molecular diagnostics and precision oncology, treatment paradigm for lung cancer have shifted from histology-based approaches to biomarker-driven strategies, substantially improving outcomes for patients with NSCLC (9). Third-generation EGFR-TKIs, represented by osimertinib, have become the standard first-line treatment for patients with *EGFR*-mutated advanced NSCLC, offering substantial survival benefits and superior efficacy against CNS metastases (10). However, acquired resistance to EGFR-TKIs remains a critical challenge, limiting long-term efficacy. Resistance mechanisms are broadly classified into on-target and off-target resistance. Among these, acquired oncogenic driver switching—particularly via kinase fusions—represents a rare but clinically important resistance pattern. In NSCLC, *EGFR* mutations and *ALK* rearrangements are generally considered mutually exclusive driver alterations (11, 12). Nevertheless, recent evidence suggests that treatment pressure exerted by EGFR-TKIs may lead to the emergence of rare bypass resistance mechanisms mediated by ALK fusions (6–8). In a large-scale retrospective study involving 3,505 patients with *EGFR*-mutated NSCLC, only 31 patients (0.9%) were found to harbor concurrent kinase fusions, of whom 12 were confirmed to have newly acquired fusions post-treatment (13). The uniqueness of this case lies in the emergence of an exceptionally rare dual bypass activation pattern following a remarkable five-year period of disease control on first-line osimertinib. Notably, the nearly five-year disease control achieved with first-line osimertinib is itself clinically uncommon, particularly in the presence of a concurrent *TP53* mutation, which has generally been associated with poorer outcomes and shorter duration of benefit from EGFR-TKI therapy in *EGFR*-mutated NSCLC. This prolonged response further highlights the exceptional clinical trajectory of the present case. Upon disease progression, longitudinal re-biopsy revealed the simultaneous acquisition of an *EML4-ALK* fusion and a *BRAF* V600G mutation, accompanied by the complete loss of the primary *EGFR* mutation. This finding suggests that the recurrent tumor was no longer primarily dependent on the original *EGFR*-driven clone. Instead, the emergence of an *EML4-ALK* fusion with relatively high VAF and concordant ALK positivity by immunohistochemistry supports a model of molecularly distinct clonal replacement or oncogenic driver switching under prolonged EGFR-TKI selective pressure. This pattern differs from conventional EGFR-TKI resistance, in which the original *EGFR* mutation is usually retained and resistance is mediated by secondary *EGFR* alterations or bypass pathway activation. Nevertheless, we cannot fully exclude the possibility that a low-frequency *ALK*-positive subclone existed below the detection threshold at baseline and was subsequently selected during long-term osimertinib therapy. While previous studies have indicated that prolonged selective pressure from EGFR-TKIs can induce *ALK* fusions or *BRAF* V600E mutations (14), the concurrent acquisition of an *EML4-ALK* fusion and a *BRAF* V600G mutation remains unprecedented in the existing literature. Furthermore, the clinical significance of the atypical *BRAF* V600G variant in the context of acquired resistance remains largely unexplored.

The coexistence of an *EML4-ALK* fusion and a *BRAF* V600G mutation posed a therapeutic challenge. In this case, monotherapy with lorlatinib was selected based on several considerations. First, the VAF of the *EML4-ALK* fusion was substantially higher than that of the *BRAF* V600G mutation (31.96% vs. 13.57%), suggesting that the ALK pathway was the dominant oncogenic driver and principal contributor to disease progression. Second, lorlatinib, a third-generation ALK-TKI, has broad activity against ALK resistance mutations and excellent intracranial penetration, with a reported cerebrospinal fluid-to-plasma concentration ratio approaching 0.96, which is particularly relevant for patients with established brain metastases (15, 16). Third, previous case reports of tumors harboring concurrent *ALK* fusions and *BRAF* V600E mutations have shown clinically meaningful responses to ALK inhibitors in selected cases (17), suggesting that the *ALK* fusion may represent the dominant actionable driver in certain co-altered tumors. However, this should not be interpreted as direct activity of ALK inhibitors against *BRAF*-mutant clones. The favorable thoracic response observed in this patient supports the therapeutic relevance of targeting the predominant driver event, namely the *ALK* fusion. Nevertheless, the intracranial response should be interpreted cautiously, as CyberKnife SRS had been administered before lorlatinib initiation. Thus, the subsequent reduction in the brain lesion may reflect the combined effects of prior local radiotherapy and systemic ALK inhibition and cannot be definitively attributed to lorlatinib alone. In contrast, the *BRAF* V600G mutation may represent a concomitant resistant subclone or a passenger alteration, and its capacity to activate the MAPK pathway, as well as its therapeutic actionability, remains uncertain. In the present case, the VAF of *BRAF* V600G was lower than that of the *EML4-ALK* fusion, and the patient achieved marked thoracic regression after ALK inhibition. These findings suggest that *BRAF* V600G may represent a concomitant resistant subclone or passenger-like alteration rather than the predominant driver at the time of progression. Serial plasma ctDNA monitoring, if clinically feasible, may be useful in future follow-up to track the clonal dynamics of the *BRAF* V600G alteration and determine whether this subclone expands under ALK inhibition, although routine implementation may be limited by cost and accessibility. The optimal treatment strategy for patients with concurrent *EGFR* and *ALK* alterations remains controversial. Some studies have suggested that dual TKI therapy with an EGFR-TKI plus an ALK-TKI may be more effective (18), whereas other case reports have shown that ALK inhibitor monotherapy can induce more durable and profound responses than EGFR-TKI therapy (19). In the present case, the disappearance of the original *EGFR* mutation suggested loss of dependence on the founding *EGFR*-driven clone, thereby obviating the need for dual EGFR/ALK inhibition and simplifying therapeutic decision-making.

This case highlights several critical implications for the clinical management of NSCLC, particularly in the context of EGFR-TKI resistance. First, it underscores the critical need for longitudinal repeat biopsies. Particularly following prolonged EGFR-TKI therapy and upon CNS progression, re-biopsy remains the definitive method to uncover rare resistance mechanisms. Second, it demonstrates the value of comprehensive genomic profiling via NGS to simultaneously detect diverse genetic alterations, thereby preventing the under-recognition of complex co-mutations. Third, it illustrates the importance of accurately identifying the dominant oncogenic driver. In scenarios with concurrent mutations, integrating the VAF with clinical and literature evidence is essential to pinpoint the primary driver and tailor targeted therapy. Finally, this case serves as a compelling reminder of the profound genomic instability that can manifest as polyclonal resistance following osimertinib failure, as evidenced by the concurrent acquisition of *ALK* and *BRAF* alterations. While this case demonstrates successful management of dual bypass resistance through lorlatinib monotherapy, several challenges remain. The relatively short follow-up duration after lorlatinib initiation limits the ability to assess long-term efficacy, tolerability, and the potential emergence of additional resistance mechanisms. If disease progression occurs again, repeat biopsy and/or plasma-based comprehensive genomic profiling should be prioritized to reassess the dominant oncogenic driver. Potential scenarios include ALK-dependent resistance, MAPK pathway-driven progression, histologic transformation, or polyclonal resistance. If ALK-dependent progression emerges, subsequent ALK-directed therapeutic strategies or clinical trial enrollment may be considered. Conversely, if the *BRAF* V600G subclone expands or MAPK pathway activation becomes dominant, BRAF/MEK-directed therapy or rational combination approaches targeting both ALK and BRAF pathways may warrant consideration. However, the optimal therapeutic strategy for such complex resistance scenarios remains undefined and requires further investigation.

## 4 Conclusion

This case represents the first reported instance of a rare dual bypass activation resistance pattern involving acquired *EML4-ALK* fusion with *BRAF* V600G mutation following prolonged first-line osimertinib treatment in *EGFR*-mutant lung adenocarcinoma. Through repeat biopsy and comprehensive molecular profiling, the *ALK* fusion was successfully identified as the predominant actionable driver. Subsequent monotherapy with the third-generation ALK-TKI lorlatinib was associated with rapid and marked regression of thoracic lesions, while reduction of the previously irradiated intracranial lesion was also observed. This case emphatically underscores that longitudinal, biopsy-based molecular profiling upon targeted therapy resistance remains the absolute cornerstone of precision oncology. Furthermore, when navigating such exceedingly rare concurrent alterations, deciphering the dominant oncogenic dependency is paramount for rational therapeutic selection. Ultimately, this report provides an invaluable clinical blueprint for managing hyper-complex targeted therapy resistance scenarios in practice.

## Patient perspective

Five years ago, when I was initially diagnosed with advanced lung cancer, I was completely overwhelmed and terrified. However, taking the daily targeted medication (osimertinib) felt like a miracle; it allowed me to regain my normal life without experiencing any noticeable side effects. Recently, when my condition worsened, my anxiety returned, and I deeply feared that I had run out of treatment options. Throughout this, my oncology team was incredibly supportive. They clearly explained the necessity of undergoing CyberKnife radiosurgery and a repeat biopsy. Learning that my tumor had changed and developed a ‘new’ target was surprising, but it ultimately brought me a renewed sense of hope. Since transitioning to the new medication (lorlatinib), my cough and chest tightness have disappeared, and I feel significantly more energetic. I am profoundly grateful for the advanced genetic testing and personalized care I have received, which have given me the confidence to continue fighting this disease.

## Data Availability

The original contributions presented in the study are included in the article/supplementary material. Further inquiries can be directed to the corresponding author.

## References

[1] National Comprehensive Cancer Network . NCCN Clinical Practice Guidelines in Oncology: non-Small Cell Lung Cancer Version 3. 2026 (2025). Available online at: https://www.nccn.org/guidelines (Accessed March 30, 2026).

[2] Bouchard N Daaboul N . Lung cancer: targeted therapy in 2025. Curr Oncol. (2025) 32:146. doi: 10.3390/curroncol32030146 40136350 PMC11941068

[3] Kobayashi Y Azuma K Nagai H Kim YH Togashi Y Sesumi Y . Characterization of EGFR T790M, L792F, and C797S mutations as mechanisms of acquired resistance to afatinib in lung cancer. Mol Cancer Ther. (2017) 16:357–64. doi: 10.1158/1535-7163.MCT-16-0407 27913578

[4] Xu CW Wang WX Huang RF He C Liao XH Zhu YC . Patient harboring a novel PIK3CA point mutation after acquired resistance to crizotinib in an adenocarcinoma with ROS1 rearrangement: a case report and literature review. Thorac Cancer. (2017) 8:714–9. doi: 10.1111/1759-7714.12496 28845578 PMC5668514

[5] Chen Y He M Dai Z Wang Y Chen J Wang X . Clinical and molecular profiling of EGFR-mutant lung adenocarcinomas transformation to small cell lung cancer during TKI treatment. Front Oncol. (2023) 13:1308313. doi: 10.3389/fonc.2023.1308313 38188289 PMC10770233

[6] Cheung BMF El Helali A Leung DKC . Anaplastic lymphoma kinase (ALK) fusion as a resistance mechanism to epidermal growth factor receptor (EGFR) tyrosine kinase inhibitors with complete response after osimertinib and alectinib: a case report and literature review. Cureus. (2025) 17:e92202. doi: 10.7759/cureus.92202 41089107 PMC12516796

[7] Yin Q Guo T Zhou Y Sun L Meng M Ma L . Effectiveness of alectinib and osimertinib in a brain metastasized lung adenocarcinoma patient with concurrent EGFR mutations and DCTN1-ALK fusion. Thorac Cancer. (2022) 13:637–42. doi: 10.1111/1759-7714.14291 34964276 PMC8841708

[8] Nguyen HV Do CH Tran BT Trinh HL . Rare sequential EGFR and ALK mutations in non-small cell lung cancer: a case report and literature review. Oncol Lett. (2025) 30:398. doi: 10.3892/ol.2025.15144 40606307 PMC12215245

[9] Abdelmonem A Bikram Shah B Shebli MA Tabbara IA . Targeted therapies in non-small cell lung cancer: a contemporary review. Am J Clin Oncol. (2025). doi: 10.1097/COC.0000000000001275 41396253

[10] Lu S Dong X Jian H Chen J Chen G Sun Y . AENEAS: a randomized phase III trial of aumolertinib versus gefitinib as first-line therapy for locally advanced or metastatic non-small-cell lung cancer with EGFR exon 19 deletion or L858R mutations. J Clin Oncol. (2022) 40:3162–71. doi: 10.1200/JCO.21.02641 35580297 PMC9509093

[11] Soda M Choi YL Enomoto M Takada S Yamashita Y Ishikawa S . Identification of the transforming EML4-ALK fusion gene in non-small-cell lung cancer. Nature. (2007) 448:561–6. doi: 10.1038/nature05945 17625570

[12] Wang J Dong Y Cai Y Zhou L Wu S Liu G . Clinicopathologic characteristics of ALK rearrangements in primary lung adenocarcinoma with identified EGFR and KRAS status. J Cancer Res Clin Oncol. (2014) 140:453–60. doi: 10.1007/s00432-014-1584-8 24442099 PMC11824140

[13] Schrock AB Zhu VW Hsieh WS Madison R Creelan B Silberberg J . Receptor tyrosine kinase fusions and BRAF kinase fusions are rare but actionable resistance mechanisms to EGFR tyrosine kinase inhibitors. J Thorac Oncol. (2018) 13:1312–23. doi: 10.1016/j.jtho.2018.05.027 29883838

[14] Zeng Y Zeng Q Yang B Hu Y . Therapeutic strategies to overcome ALK-fusion and BRAF-mutation as acquired resistance mechanism in EGFR-mutated non-small cell lung cancer: two case reports. Front Oncol. (2024) 14:1390523. doi: 10.3389/fonc.2024.1390523 39555453 PMC11563980

[15] Wang L Wang W . Safety and efficacy of anaplastic lymphoma kinase tyrosine kinase inhibitors in non-small cell lung cancer (review). Oncol Rep. (2021) 45:13–28. doi: 10.3892/or.2020.7851 33200229 PMC7709813

[16] Wu YL Kim HR Soo RA Zhou Q Akamatsu H Chang GC . First-line lorlatinib versus crizotinib in Asian patients with advanced ALK-positive NSCLC: five-year outcomes from the CROWN study. J Thorac Oncol. (2025) 20:955–68. doi: 10.1016/j.jtho.2025.02.021 40024442

[17] Guo W Liang J Zhang D Huang X Lv Y . Lung adenocarcinoma harboring complex EML4-ALK fusion and BRAF V600E co-mutation responded to alectinib. Med (Baltimore). (2022) 101:e30913. doi: 10.1097/MD.0000000000030913 36221356 PMC9542562

[18] Ren KH Qin WW Wang Y Peng JC Hu WX . Detection of an EML4-ALK fusion mutation secondary to epidermal growth factor receptor-tyrosine kinase inhibitor (EGFR-TKI) therapy for lung cancer: a case report. Ann Palliat Med. (2022) 11:2503–9. doi: 10.21037/apm-22-744 35927783

[19] Chen Y Qi F Zeng Y Tan J Hu M Zhang H . A case of lung adenocarcinoma with concurrent EGFR mutation and ALK fusion combined with literature review. Clin Case Rep. (2026) 14:e71749. doi: 10.1002/ccr3.71749 41497773 PMC12765661

